# From vulnerability to resilience: Understanding social responses to climate-induced hazards in peri-urban and natural resources-dependent communities – The case of Tanga City in Tanzania

**DOI:** 10.4102/jamba.v18i1.2040

**Published:** 2026-09-14

**Authors:** Given J. Mhina, Tumpale Sakijege, Dionis S. Rugai, Neema Kijazi

**Affiliations:** 1 Department of Environmental Science and Management, School of Engineering and Environmental Studies, Ardhi University, Dar es Salaam, Tanzania; 2 Department of Urban and Regional Planning, School of Spatial Planning and Social Sciences, Ardhi University, Dar es Salaam, Tanzania; 3 Institute of Building Research, Ardhi University, Dar es Salaam, Tanzania

**Keywords:** livelihood security, adaptation strategies, community vulnerability, co-adaptation, climatec change

## Abstract

Over the past two decades, climate change (CC) has been and continues to be considered among the greatest challenges of our time. Its escalating risks are increasingly evident and more intense, particularly in natural resources-dependent and rapidly growing communities of sub-Saharan Africa. This study examined the interplay of climate-induced hazards, social vulnerability, and adaptation strategies used among communities in peri-urban areas of Tanga City in Tanzania. The study objectives were achieved through a mixed methods approach, in which household surveys, stakeholder meetings, and community design workshops were the main data collection methods. Findings indicate that prolonged dry spells, extreme temperatures, and outbreaks of waterborne diseases were recognised among the most severe CC-related hazards. Floods and saltwater intrusion showed more variability in perception due to localised exposure. Livelihoods – like small-scale urban agriculture and mariculture (which includes seaweed cultivation, sea cucumber farming and sea salt production) – were identified as extremely vulnerable to CC, yet crucial for community resilience. Responding to climate variability, various adaptation strategies have emerged, ranging from livelihood diversification to household-level climate-sensitive resource management.

**Contribution:**

The findings underscore the importance of integrating indigenous knowledge, socio-ecological diversity and stakeholder participation in the assessment and management of climate risks. The research emphasises the need for context-specific adaptation planning focused on community engagement, nature-based strategies and improved institutional capabilities. It contributes to limited empirical evidence on peri-urban climate vulnerability and emphasises the urgency of mainstreaming climate risk management into local development frameworks through participatory and inclusive governance.

## Introduction

Climate change (CC) stands among the most challenging environmental issues of our time. It contributes to many humanitarian emergencies, including heatwaves, wildfires, floods, tropical storms, and hurricanes, many of which increase in scale, frequency and intensity. Research indicates that 3.6 billion people already live in areas that are highly vulnerable to CC, and projections suggest that this number will continue to increase in the coming decades (WHO 2023). It is evident from the literature that CC affects our life-supporting ecosystems, economies and communities in many different ways (Reyes-García et al. 2023; Tol 2018; Verma 2021). Extreme weather events such as floods, droughts and storms have become more frequent, threatening lives, livelihoods and ecosystems (Legg 2021).

In most cases, CC vulnerability is context-specific. Peri-urban and natural resource-dependent communities are particularly more vulnerable to extreme weather events due to their geographical location, economic dependence on climate-sensitive resources and limited access to adaptation support (Carrilho & Trindade 2022; Yanda et al. 2019). Peri-urban areas are transitional zones between rural and urban environments where, in the context of the Global South, most such areas are characterised by rapid population growth, changing land use and uneven access to infrastructure and services (Carrilho & Trindade 2022). Moreover, natural resource-dependent communities refer to populations heavily reliant on climate-sensitive natural resources, making them particularly sensitive to climate variability and extreme weather events. These communities often struggle to recover from disasters, making them highly susceptible to climate shocks (Fischer 2018).

In contrast to vulnerability, resilience refers to the capacity of individuals, households or communities to absorb shocks, adapt to changing conditions and recover from disturbances while maintaining essential functions and livelihoods (Rubekie, Pauline & Kaaya 2022; Yanda et al. 2019). Resilience is shaped by factors such as access to assets, social networks, institutional support, knowledge systems and adaptive strategies. While social vulnerability plays a critical role in determining the extent to which a community is impacted by climate-induced hazards, resilience is defined by how a community experiences and recovers from climate risks and is often a function of socio-economic status, infrastructure robustness and access to coping resources (Deziel et al. 2023; Mah et al. 2023). Peri-urban and natural resource-dependent communities face unique challenges in disaster risk preparedness and response (Carrilho & Trindade 2022). Without effective risk reduction strategies, these populations remain trapped in cycles of vulnerability and loss.

As climate-induced hazards escalate, affected communities adopt various short-term coping mechanisms and long-term adaptation strategies to cope with the rapidly changing climate (Darjee, Neupane & Köhl 2023). Traditional coping strategies include mechanisms like migration, diversifying livelihoods and reliance on social networks for support (Dapilah, Nielsen & Friis 2020; Hossain et al. 2024). However, not all coping mechanisms are sustainable, and some, such as overexploitation of natural resources and environmental migration, may exacerbate long-term environmental and social vulnerabilities (Bharadwaj et al. 2025; Szaboova et al. 2023). Adoption of effective adaptation strategies, such as climate-smart agriculture, improved infrastructure, and early warning systems, is widely emphasised and will continue to be essential in strengthening social and environmental resilience to reduce long-term risk exposure (Ariom et al. 2022; Okoronkwo et al. 2024).

Building resilience to climate risks is crucial in enabling active community involvement in the transition from vulnerability to long-term environmental and social sustainability (Elmqvist et al. 2019; Naqvi 2024). Resilience to climate change has many dimensions; it involves not only the ability to recover from climate-induced disasters, but also the capacity to anticipate and adapt to future climate-related risks (Camacho et al. 2023; Charmes 2019). Among others, the main factors that contribute to climate resilience include but are not limited to having strong social networks, equitable access to information and technology, responsive governance and climate-resilient infrastructure (Chien, Hori & Saito 2022). Without proactive resilience-building efforts, communities remain at risk of repeated climate shocks and economic instability (Bowen et al. 2020; Ma, Qirui & Lv 2023).

Despite growing research interest on adapting vulnerable communities to impacts climate change, knowledge gaps remain in understanding localised social responses to climate hazards, particularly in peri-urban, natural resource-dependent settings. Most studies focus on rural or urban areas, often overlooking the unique challenges and opportunities that exist at the urban–rural interface (Laband & Lockaby 2020; Ortiz-Báez, Cabrera-Barona & Bogaert 2021). Using peri-urban areas and resource-dependent communities of Tanga City, this study aims to assess the nature of CC–induced hazards in peri-urban communities, with specific emphasis on understanding how such hazards influence household livelihoods, shape social vulnerability and stimulate local adaptation responses.

## Research methods and design

As highlighted earlier, this study was conducted in the peri-urban areas of Tanga City, the second most populated seafront city and the fifth largest urban centre in Tanzania, located on the northeastern coast of the country along the Indian Ocean. It lies between longitudes 38°53′ and 39°10′ East and latitudes 5°00′ and 5°16′ South (Figure 1). It is characterised by a low-lying coastal terrain‌ with mangrove-lined shorelines undulating gently to the mainland. Influenced by its proximity to the Indian Ocean, the city experiences a bimodal rainfall pattern with annual rainfall ranging between 1200 mm and 1500 mm and average monthly temperatures between 24 °C and 32 °C. These conditions make Tanga City highly susceptible to climate-induced hazards. Administratively, the city is divided into 27 wards, with an estimated population of approximately 450 420 residents. For the purpose of this study, 14 wards were purposefully selected based on their historical exposure to climate-related events and the community’s economic reliance on climate-sensitive natural resources (Asari et al. 2021).

**FIGURE 1:**
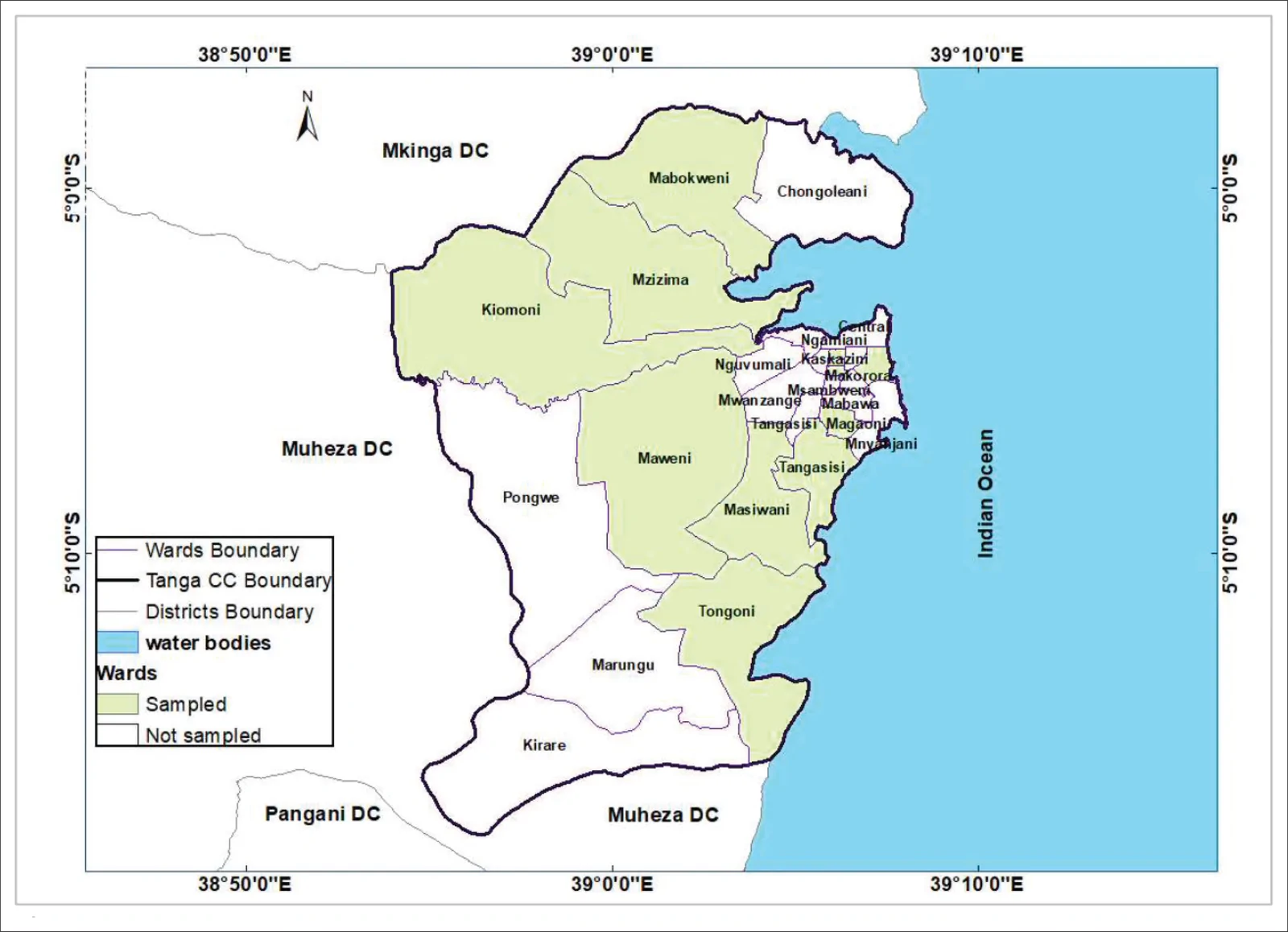
Tanga City map indicating the location of sampled wards.

### Data collection methods

To achieve the objectives of the study, both quantitative and qualitative methods were adopted to attain complementary evidence of the needed information. The primary data collection methods were applied sequentially, beginning with household surveys, followed by stakeholders’ meetings, and community design workshops. This order was maintained such that insights generated at each stage informed and complemented the subsequent phase of data collection. Supplementary methods like field investigations and analysis of satellite images were also adopted to enable an in-depth understanding of the study area and selection of representative case sites.

The combination of the methods was designed to capture both measurable patterns of hazard exposure and the lived experiences of affected communities. Quantitative data were collected through structured household surveys using Likert scales to assess community perceptions of hazard severity, while qualitative data were obtained from community design workshops and stakeholder discussions intended to explore CC coping mechanisms and livelihood adaptations adopted by the study community.

### Household survey

A household survey using a structured questionnaire was employed to examine how communities in Tanga City perceive CC, how it affects their livelihoods and the coping mechanisms they adopt. A multi-stage sampling strategy was adopted. In the first stage, 14 sub-wards were purposively selected to capture the diversity of socio-environmental settings within peri-urban Tanga, particularly areas with varying exposure to climate-related risks. In the second stage, households in each selected sub-ward were identified using the knowledge of local government leaders and systematically sampled to ensure broad spatial coverage and reduce selection bias. A total of 4850 households, representing 14% of all households in peri-urban Tanga, were surveyed. Among the respondents, 63.1% were male, and 36.9% were female, with ages ranging from 36 years to 78 years. Questionnaires were administered face‍-to-face by 25 pre-trained enumerators to ensure consistency and accuracy of data collection. Respondents were either household heads or primary household caretakers, as they are more likely to provide reliable information on household characteristics and decision-making processes. Households with temporary residents were excluded from the study. Ethical considerations, including informed consent and the confidentiality of respondents, were strictly upheld throughout the study. The survey was conducted over a period of 4-weeks, and all responses‌ were digitised, cleaned, and analysed using Microsoft Excel.‍ Although purposive selection of sub-wards might have limited the statistical generalisability of the findings it was appropriate for ensuring the inclusion of climate-vulnerable contexts relevant to the study objectives.

### Stakeholders’ meetings

As a supplementary method for data collection, one stakeholders’ meeting was organised to gather insights from selected decision-makers, utility providers, development partners and institutional representatives in Tanga City. This meeting was informed by initial insights from the household survey and meant to understand stakeholders’ views on CC, its impact on their institutional line of duties and the strategies used to address such challenges. Participants were purposefully selected based on their roles in local governance, urban planning and environmental management. Categorically, 27 stakeholders were invited, three from NGOs, two from community-based organisations, four from local government officials at ward and sub-ward levels, three from department heads from the Tanga City Council, two delegates from utility agencies and 13 from local community representatives, including youth, elders and those with special needs. The discussions followed a structured format, and key takeaways were recorded for analysis along with the household-level data.

### Community design workshops

Building on preliminary findings from the household survey and stakeholders, a modified community design charrette, guided by Lennertz and Lutzenhiser (2006), was conducted to collect additional data and triangulate household-level insights. The workshop convened 78 participants, drawing representatives from municipal officials (10), community members (42), local government leaders (14), and non-governmental organisations (12) from within Tanga City. The main objective of the workshop was to co-design a context-specific and implementable climate change (CC) adaptation plan. Specifically, participants engaged in focus group activities to identify and rank CC-induced hazards according to perceived severity using a 1–5 Likert scale (1 = most severe) and collaboratively developed contextual adaptation measures informed by local knowledge and lived experience. Additionally, the workshop was moderated in a manner that the reliability and relevance of the collected data are enhanced.

### Ethical considerations

Ethical clearance to conduct this study was obtained from the Ardhi University Research Ethics Committee (Ref. No. AE.297/327/07).

## Results and discussion

### Climate change-induced hazards affecting communities in peri-urban Tanga

The analysis results of the most popular CC-induced hazards affecting peri-urban communities in Tanga City are presented in Figure 2. To understand the distribution and variability of responses across different factors assessed, box-and-whisker plots were used to visualise the results. With reference to Figure 2, analysis of Likert scale responses reveals significant variations in how the severity of climate-induced risks is perceived in the study community. To appreciate the exposure proneness of the community to climate-induced hazards, risk perception was clustered into three categories. The first category comprises what is perceived as the most severe hazards, as indicated by the median scores, which include prolonged dry spells, extreme temperatures, and outbreaks of water-borne diseases. These hazards consistently exhibit lower median scores, suggesting a strong consensus among respondents. Additionally, the interquartile ranges (IQRs) of these hazards are relatively narrow, indicating less variability in perceptions. This implies that most respondents rate these hazards similarly.

**FIGURE 2:**
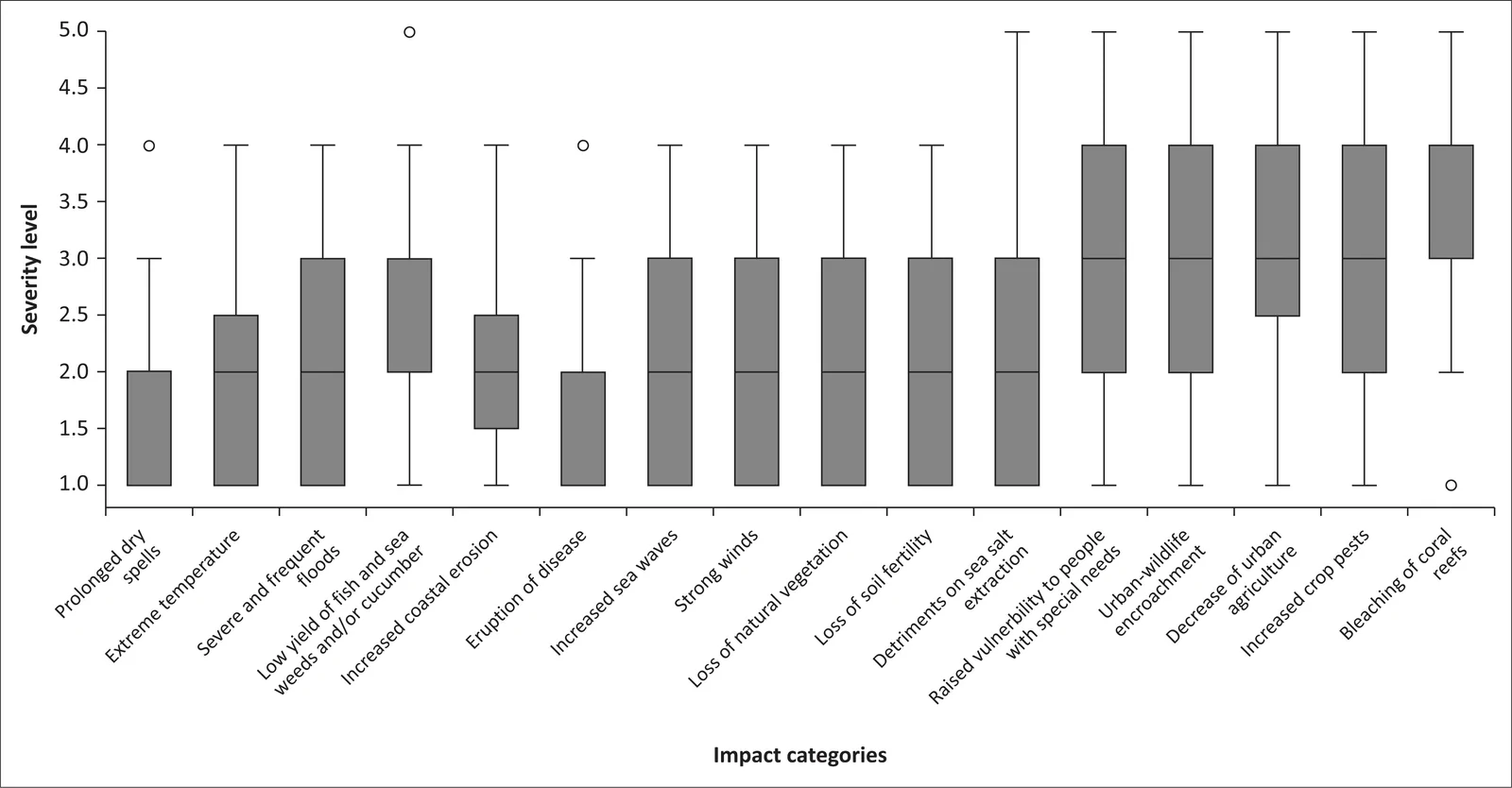
Climate-induced hazards experienced by communities living in peri-urban Tanga arranged in order of their severity.

In contrast, the second group comprises hazards with wider IQRs, many outliers and long whiskers, such as frequent floods, increased sea waves, strong winds, loss of natural vegetation and increased challenges in extracting sea salts. These are generally perceived as severe hazards by the majority of respondents, as indicated by their relatively lower median scores. However, having wide-spanning boxes reveals a weaker consensus among respondents argued from a broader divergency of opinions. Meanwhile, the third category is of hazards with higher median scores, the likes of urban-wildlife encroachment, increased vulnerability of people with special needs, and decreased produce of urban agriculture, which are perceived as hazards of moderate severity. Additionally, it was noted during the meetings with stakeholders that although these risks are perceived as moderate, they often have long-term cumulative effects that are less visible but equally significant, particularly for vulnerable groups such as women and low-income households.

In addition to survey results, insights from stakeholders’ meetings provided deeper contextual understanding of how climate risks are perceived by technocrats and bureaucrats. Among others, participants emphasised that prolonged dry spells and extreme temperatures not only affect water availability, but also constrain livelihood-supporting systems, particularly small-scale vegetable gardening, which is considered critical for many marginalised households. Moreover, insights from community design workshops and stakeholders’ meetings broadened the scope of analysis and revealed more underlying drivers of community vulnerability, including limited access to climate information, weak drainage infrastructure and limited financial capacity to adopt adaptation technologies.

As demonstrated in Figure 2, the distribution, variability and perception of CC hazards among peri-urban communities in Tanga City are in line with the emerging literature exploring the experiences of climate-induced risks in rapidly urbanising regions of sub-Saharan Africa. Observations of the current study align with findings from recent studies confirming that heat stress, drought and climate-sensitive illnesses are the most experienced and least contested CC-induced hazards among peri-urban dwellers (Ali, Pham & Xuan 2025; Lundberg 2025). According to Gemeda, Korecha and Garedew (2023) and Zhu, Zhang and Lin (2023), convergence of community risk perception is often influenced by factors like shared past experiences, frequency of exposure, socio-cultural norms and access to information, which shape how communities collectively interpret, prioritise and cope with climate risks. Homogeneity of livelihoods, geographical proximity and common adaptive challenges also contribute to aligned community perceptions, especially in frequently affected populations (Darjee et al. 2023). However, convergence in perception that transpired in the current study was mainly attributed to the recurrence and intensity or acuteness of the identified hazards, given that the majority of peri-urban communities in Tanga City are heavily reliant on climate-sensitive livelihoods with limited infrastructure resilience.

With reference to the second group of hazards exhibiting wider IQRs (Figure 2), it cannot be established with confidence that these hazards are of high priority due to the wider divergent perceptions. This variability reflects the spatial and socio-economic heterogeneity of exposure and vulnerability within peri-urban areas, as also observed in coastal and flood-prone settlements across Africa (Halecki & Bedla 2025; Hussainzad & Gou 2024; Sunjo & Fuanyi 2022). Differences in topography, land use, coastal resources dependency, and proximity to drainage infrastructure contribute to the observed divergence, particularly in hazards like flooding and saline intrusion, where impact and frequency can vary markedly even across small distances. Based on the findings, community vulnerabilities linked to category three hazards were perceived as lower-priority risks posing moderate and comparatively less severe threats. These insights align with findings that highlight a relatively low prioritisation of social, environmental and systemic risks which often occur less frequently and, to some extent, are unnoticeable, particularly in situations where more urgent climatic risks attract community attention (Haro et al. 2021; Schlosser et al. 2023). Collectively, these patterns affirm the need for climate risk assessments to account for both the physical intensity of hazards and socio-ecological heterogeneity of exposure and perception, which are normally crucial in shaping local adaptation priorities and planning efficacy (Darjee et al. 2023).

### Synergies of climate change impacts with social vulnerability and community livelihoods

The analysis of the collected data from household surveys and stakeholders’ meetings indicates that communities living in the peri-urban areas of Tanga City engage in various livelihood activities shaped by both the urban-rural interface and the region’s natural resources. Figure 3 presents a summary of livelihood activity responses provided by the respondents. Figure 3 demonstrates that while livelihoods in peri-urban Tanga are diverse, the majority of residents earn their living through employment in various sectors, whereas small-scale urban farming and mariculture rank among the top three livelihood activities, supporting more than 50% of the population.

**FIGURE 3:**
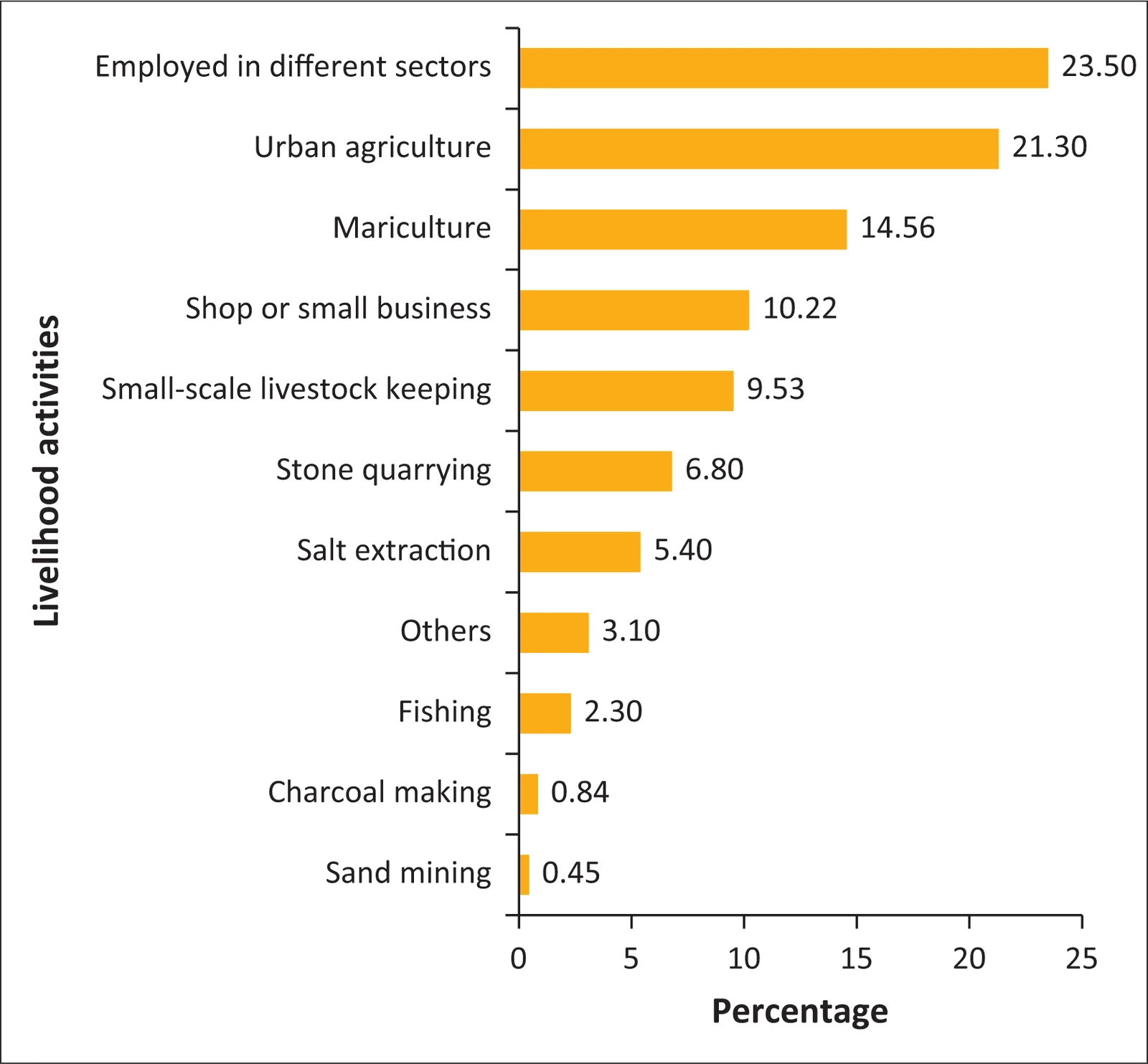
Livelihood activities of communities in the peri-urban areas of Tanga City.

While quantitative evidence identified the most dominant livelihood activities in the case study, qualitative data complemented the findings by illustrating how households, in some cases, strategically combine multiple livelihood activities to cope with environmental shocks and climate variability. Participants in stakeholder meetings emphasised that livelihood diversification is not merely an economic strategy, but also a critical adaptation mechanism that buffers households against CC impacts. Furthermore, qualitative insights revealed the presence of non-structural constraints shaping the adoption of livelihood options, including limited access to credit, land tenure insecurity, declining soil productivity and competition over coastal resources. Collectively, these factors exacerbate vulnerability to CC and constrain households’ capacity to adopt effective adaptation strategies.

With reference to Figure 3, the dominance of small-scale urban farming and mariculture as primary livelihood activities in peri-urban Tanga mirrors broader global patterns observed in similar socio-ecological settings. Recent studies indicate that peri-urban areas often serve as dynamic spaces for livelihood diversification, driven by the interplay of urban expansion and access to natural resources (Ebin 2024; Goswami & Nautiyal 2020). For example, research in the peri-urban zones of Dar es Salaam, Tanzania, highlights the role of urban agriculture in sustaining household economies and enhancing food security in response to rapid population growth and urbanisation (Ricci 2019). Similarly, in coastal regions of the Philippines, mariculture has been identified as a key adaptive livelihood, particularly for communities facing resource depletion and land-use change due to urban pressures (Fabinyi et al. 2022).

These observations are consistent with the findings of the current study, where more than 50% of residents in peri-urban Tanga rely on small-scale farming and mariculture, reflecting a global trend in which peri-urban households strategically leverage both rural and urban opportunities to build resilient livelihood systems in response to the rapidly changing urban micro-climate. It was further observed that the nature of livelihood activities in peri-urban Tanga demonstrates the synergy between local adaptive practices and broader CC resilience. The observed diversity of livelihood activities not only leverages the region’s ecological assets, but also serves as critical buffers against environmental and economic shocks. In the context of increasing climate variability, such as unpredictable rainfall patterns, sea-level rise and resource degradation, the diversification of livelihoods enhances community adaptive capacity and reduces dependence on a single income stream (Habib, Ariyawardana & Aziz 2023). Moreover, the integration of urban agriculture and mariculture within peri-urban economies contributes to food security and income stability, particularly for socially vulnerable populations, including women, youth and low-income households, who are disproportionately affected by climate-induced stressors (Lee-Smith et al. 2019). Findings from the current study suggest that fostering and supporting such hybrid livelihood systems can play a strategic role in climate adaptation planning and social protection policies, especially in rapidly urbanising regions facing intersecting environmental and socio-economic challenges.

### Climate change adaptation measures and coping mechanisms for climate-induced disasters in peri-urban communities of Tanga

Figure 4 illustrates a comparative analysis of the adoption rates of various CC adaptation measures within the study community, arranged from the least to the most utilised. The analysis reveals a clear disparity in preference, with some measures such as groundwater recharge and runoff harvesting receiving minimal adoption (1.2% and 2.0%, respectively), suggesting potential barriers such as financial constraints, technical complexity or limited awareness. In contrast, measures like afforestation (7.1%) and mangrove restoration (8.1%) show higher adoption, likely due to their perceived effectiveness, communal and ecological benefits. The prominence of nature-based solutions reflects a growing recognition of their long-term sustainability and alignment with ecosystem-based adaptation strategies. This distribution highlights the need for targeted policy interventions and capacity-building initiatives to encourage wider adoption of underutilised yet impactful adaptation measures.

**FIGURE 4:**
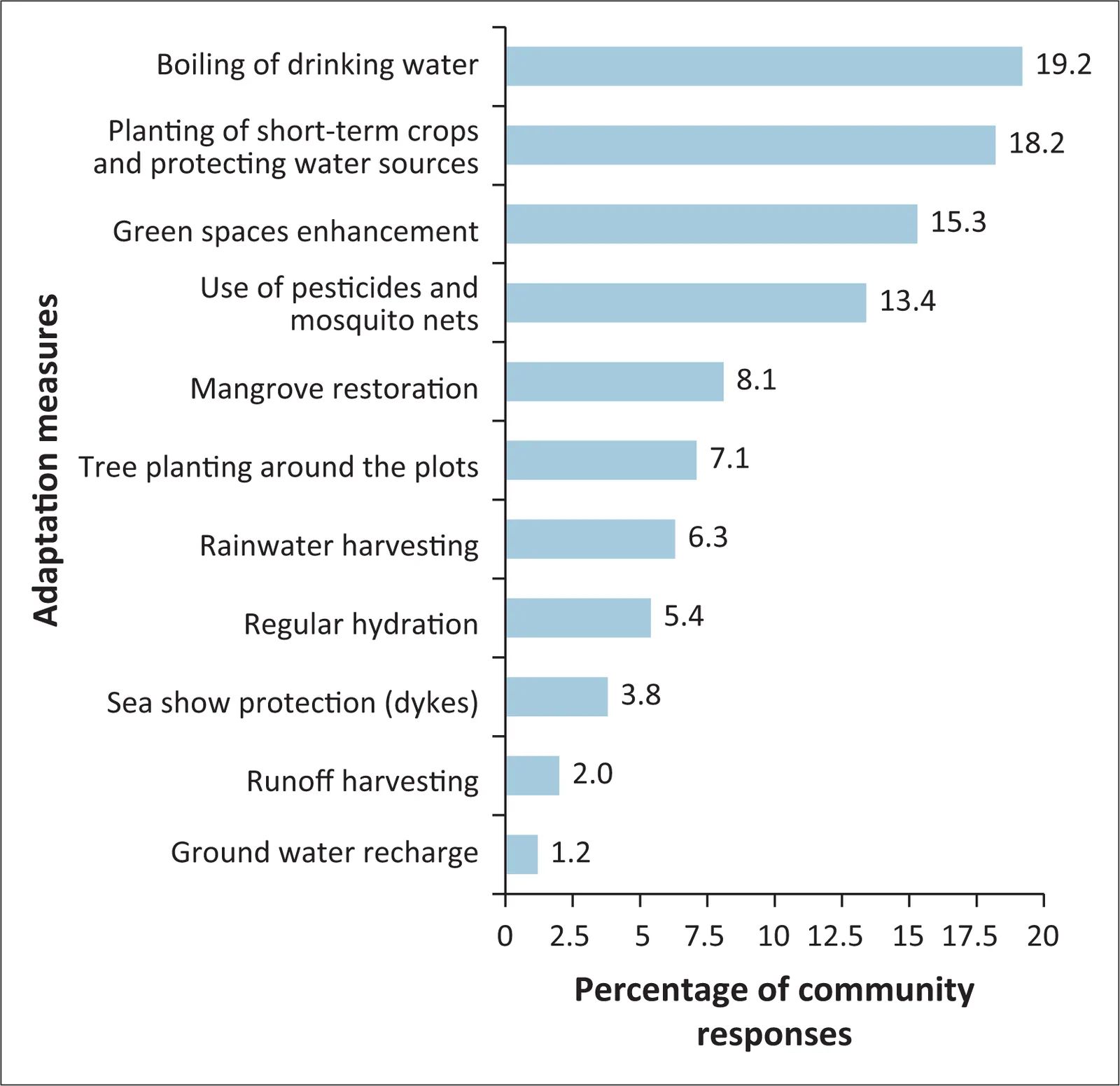
Climate change adaptation measures adopted by communities in the peri-urban areas of Tanga City.

Importantly, community design workshops moved the discussion beyond problem identification to co-development of context-specific adaptation options, including the promotion of decentralised rainwater harvesting systems, community-led mangrove restoration with livelihood incentives, and establishment of localised early warning and information-sharing mechanisms. Participants emphasised that many municipal-level initiatives aimed at mitigating CC impacts often fail due to limited community engagement, weak local ownership and insufficient public trust. Additionally, stakeholders highlighted the need to integrate local knowledge – such as seasonal indicators for rainfall prediction into CC adaptation planning and implementation. The findings of the current study are consistent with global trends observed in similar socio-ecological contexts from around the world. For example, a recent study in the Hindu Kush Himalayan region reported that the initial adoption of agroforestry was driven by risk mitigation, whereas later adoption was motivated by income diversification and ecological co-benefits (Campbell, Cameron & Subba 2025). Likewise, Ayassamy (2025) and Asari et al. (2021) highlight the increasing reliance on mangrove ecosystems for coastal protection and resilience enhancement of vulnerable coastal communities. In contrast, the relatively low adoption of rainwater harvesting and groundwater recharge techniques in this study may be attributed by technical, financial or institutional barriers, as recently documented in evaluations of sustainable water resource management strategies (Gebreslassie et al. 2025). These results emphasise the importance of policy-driven support, capacity-building initiatives and context-specific interventions to scale the implementation of effective yet underused adaptation practices.

### General reflections on climate risks, adaptation and policy gaps

Based on the presented findings, this study highlights critical insights into the dynamics of social vulnerability, climate hazards and adaptation practices in peri-urban, natural resource-dependent communities. Added to this, the integration of community-level perceptions with empirical data visualisation techniques, in this case, box and whisker plots (Figure 2) as used in the current study, contributes to understanding local climate risks and grounds the contextualisation of climate actions similar to what is accentuated by Mahyar (2024) and Zhu et al. (2023). In agreement with arguments presented by Schlosser et al. (2023), deeper interpretation of study results suggests that certain hazards tend to co-occur, reinforcing the interconnected nature of climate risks. This supports the argument that mitigation efforts should be holistic rather than isolated interventions. Notably, the classification of climate-induced hazards into categories based on their severity and variability of perceptions, enables clear prioritisation of risks in adaptation planning. This aligns with observations from other studies in sub-Saharan Africa that have emphasised the importance of community-based climate risk assessments in urbanising environments (Archer et al. 2014; Mensah et al. 2022).

Despite the analytical merits of the current study, further studies are recommended to complement the utility of the presented findings for policy and practice advancement. It was evident from this study that spatial focus of hazard exposure has the potential to drive context-specific solutions (Chien et al. 2022; Mahyar 2024). As such, for informed and targeted policy actions, future analysis should incorporate spatial mapping of hazard exposure versus the current geographic focus of adaptation measures. Additionally, spatial heterogeneity is equally crucial in shaping differential vulnerability, particularly in flood-prone or drought-affected settings (Deziel et al. 2023; Hossain et al. 2024). Incorporating geospatial techniques and hazard-specific vulnerability indices would improve the targeting of adaptation interventions and resource allocation. Furthermore, considering the dependency of the communities on natural resources, future research should explore ecosystem-based adaptation measures such as mangrove restoration, wetland conservation and agroecological practices, which are increasingly recommended as sustainable responses to CC (Ayassamy 2025; Kapuka & Hlásny 2021; Ward et al. 2016).

Policy-wise, the findings underscore the imperative of mainstreaming climate risk management into local governance frameworks and sectoral development plans within Tanga City. The study’s documentation of diversified livelihoods, including small-scale urban farming and mariculture, supports global evidence highlighting peri-urban areas as critical interfaces for livelihood adaptation in response to urbanisation and environmental stress (Carrilho & Trindade 2022; Ebin 2024; Lee-Smith et al. 2019). Strengthening local institutional capacities is thus essential for fostering resilience, particularly through improving infrastructure robustness, early warning systems and access to climate information services (Chien et al. 2022; Naqvi 2024). In addition,, fostering multi-stakeholder partnerships – including government agencies, NGOs, and private sector actors – would facilitate resource mobilisation and coordinated action. The use of formal collaboration mechanisms, such as Memoranda of Agreement (MoA), is widely advocated in climate adaptation governance to ensure accountability, clarity of roles and sustainability of interventions (Naqvi 2024). Finally, enhancing knowledge management systems to document and disseminate successful community-based adaptation practices is critical for scaling up resilience efforts across other peri-urban settings in Tanzania and beyond (Fabinyi et al. 2022; Ndesanjo & Asokan 2023).

## Conclusion

Based on empirical evidence, this study demonstrates the interconnectedness of climate-induced hazards, social vulnerability and community adaptation strategies in the peri-urban, natural resource-dependent communities. By examining what enhances resilience to CC from the perspective of community-level risk perception, the study findings not only highlight the diverse and context-specific nature of climate risks, but also underscore the adaptive capacities embedded in localised community livelihood systems. While the study has advanced methodological approaches for assessing climate risks using community perceptions, further integration of spatial vulnerability analysis and ecosystem-based adaptation measures is necessary to strengthen future research and practice. Importantly, the study emphasises the need to mainstream climate risk management into local development planning, supported by multi-stakeholder collaboration, institutional capacity strengthening and knowledge-sharing platforms. These policy reflections align with emerging global and regional discourses advocating for inclusive, participatory and nature-based solutions to climate resilience. Ultimately, fostering localised, multi-dimensional adaptation strategies will be critical for enhancing long-term resilience and sustainability of peri-urban communities facing escalating climate-related challenges.
